# A comparative study of industry responses to government consultations about alcohol and gambling in the UK

**DOI:** 10.1093/eurpub/ckad018

**Published:** 2023-02-28

**Authors:** Saloni Bhuptani, Sadie Boniface, Katherine Severi, Greg Hartwell, Elizabeth McGill

**Affiliations:** LSHTM, Faculty of Public Health and Policy, London, UK; Institute of Alcohol Studies, London, UK; Addictions Department, Institute of Psychiatry, Psychology & Neuroscience, King’s College, London, UK; Institute of Alcohol Studies, London, UK; LSHTM, Faculty of Public Health and Policy, London, UK; LSHTM, Faculty of Public Health and Policy, London, UK

## Abstract

**Background:**

There is growing evidence that common strategies are used across unhealthy commodity industries (UCIs) to influence policy decisions in line with their commercial interests. To date, there have been relatively few studies comparing corporate political activity (CPA) across UCIs, especially comparing the alcohol and gambling industries.

**Methods:**

A comparative and inductive thematic analysis of alcohol and gambling industry submissions to two House of Lords (HoL) inquiries in the UK was conducted. Themes in the framing, arguments and strategies used by the alcohol and gambling industries in CPA were compared.

**Results:**

Alcohol and gambling industry responses largely used the same framings, both in terms of the problems and solutions. This included arguing that harms are only experienced by a ‘minority’ of people, emphasising individual responsibility and shifting blame for harms to other industry actors. They promoted targeted or localised solutions to these harms, in place of more effective population level solutions, and emphasised the perceived harms of introducing regulation not in the industries’ interests.

**Conclusions:**

These findings are consistent with previous literature suggesting that UCIs use the same framing and arguments to shape the narrative around their harms and solutions to those harms. This study also identified novel strategies such as shifting blame of harms to other industry actors. Policy makers should be aware of these strategies to avoid undue industry influence on policy decisions and understanding commonalities in strategies may help to inform more effective public health responses across all UCIs.

## Introduction

Consumption of products produced by unhealthy commodity industries (UCIs), e.g. the tobacco, alcohol, ultra-processed food and increasingly, gambling industries represent important public health concerns.^1^

Regulation of UCIs has proven complex, at least in part due to corporate political activity (CPA), the way in which corporations use their power to influence policy decisions to secure a policy environment conducive to consumption of their products.^2^ In policy making, CPA can be used as means to ‘frame’ harms from UCIs in industries’ favour^3^ and can serve to prevent, delay or weaken policies that are in the interest of public health, undermine research and capture the public debate in line with commercial interests.^2^ There has been considerable research into the CPA of the tobacco industry. Attention has turned to other UCIs, such as the alcohol industry,^4^ and more recently, the gambling industry,^5^ although there has been considerably less research conducted on the latter.

While alcohol and gambling differ in many ways, there are important parallels: they are common in modern society, can lead to health harms, represent major public health concerns^1^ and have a generally ‘liberal’ set of laws governing them.^6^ In England, there are approximately 602 391 dependent drinkers (2018/19)^7^ and about 245 600 people experiencing ‘problem gambling’ (2018). The number of people who experience harms from gambling is similar to the number of people who experience issues with illicit drugs.^6^ Despite discourse centring around acute harms for ‘problem’ gamblers and those who ‘misuse’ alcohol, harms from alcohol and gambling can be long lasting and extend far beyond the individual. Both have wider impacts on families, communities and society. Both can lead to crime, violence, family and social relationship breakdown and financial impacts on dependents.^8^^,^^9^

Analyses of the tobacco and alcohol industries suggests that UCIs use common strategies to subvert effective harm reduction policies. These strategies include: (i) distorting or misusing evidence,^3–5^^,^^10–12^ (ii) making unsubstantiated claims about unintended consequences of potential regulations,^10–12^ (iii) emphasising corporate social responsibility activities^5^^,^^10^^,^^12^ and (iv) misusing scientific concepts such as complexity.^12^^,^^13^ These tactics serve to frame harms as only experienced by a minority of consumers, frame the industry as part of the solution and promote industry-preferred non-regulatory activities in place of more effective population level policies.

Maani et al.^14^ suggested that understanding the CPA of UCIs is limited by the fact that most research is industry-specific, calling for more cross-sector or comparative research to understand the common strategies used by UCIs. This approach holds potential to apply learnings from the alcohol and tobacco industries to the gambling industry and to build a consistent approach across UCIs to tackle harms.^1^ However, there are very few studies comparing CPA across UCIs and especially few comparing the alcohol and gambling industries. Therefore, this study aimed to compare alcohol and gambling industry arguments used in responses to two recent House of Lords (HoL) inquiries.

## Methods

Thematic analysis^15^ was used to compare responses from the alcohol industry to the HoL inquiry into the Alcohol ‘Licensing Act 2003: post-legislative scrutiny’ with gambling industry responses to the HoL inquiry into the ‘Social and Economic Impact of the Gambling Industry’ to identify common and diverging arguments and frames used by industries in CPA. Responses to the alcohol and gambling inquiries can be viewed at https://old.parliament.uk/licensing-act-committee/ and https://committees.parliament.uk/committee/406/gambling-industry-committee/respectively.

Both inquiries followed a similar format and gathered insight into successes and shortcomings of current Alcohol and Gambling Acts in England (the Alcohol ‘Licensing Act 2003’ and the ‘Gambling Act 2005’ and related regulation). Both acts represent a liberalisation of the rules regulating the alcohol and gambling industries,^6^ making them suitable for comparison.

For each inquiry, relevant industry submissions were downloaded from the UK Parliament website. Submissions from the alcohol or gambling industry were identified based on the inclusion/exclusion criteria in tables 1 and 2 respectively. Inclusion/exclusion criteria are based on the World Health Organisation definition of the alcohol industry (which was then adopted by Public Health England).^16^ We adapted this definition for the gambling industry, as we could not find a formally agreed definition of that industry. Definitions can be found in Supplementary file S1.

**Table 1:** Inclusion and exclusion criteria for alcohol industry submissions

Inclusion	Exclusion
Types of responses	Types of responses
Written responses to the inquiry (first submission only)	Supplementary evidence
	Oral evidence
Responses from	Responses from
Manufacturers of alcohol	NGOs
Wholesale distributors of alcohol	Individuals (with no tie to the industry)
Retailers on-trade (e.g. pubs and clubs)	Local/governmental authorities
Retailers off-trade (e.g. newsagents and supermarkets)	Regulators
Importers of alcohol	Academics
Social aspects and public relations organisations (SAPROs) (e.g. charities funded by the industry)	Licensing firms/consultants^a^
Trade associations	Any other entities falling outside the alcohol industry definition given in the methods

a Licensing firms/consultants have been omitted because in addition to representing industry, these firms will also represent local authorities and the police; therefore, it is very difficult to tease out to what extent they are representing industry interests in their responses.

**Table 2:** Inclusion and exclusion criteria for gambling industry submissions

Inclusion	Exclusion
Types of responses	Types of responses
Written responses to the inquiry (first submission only)	Supplementary evidence
	Oral evidence
Responses from	Responses from
On-shore operators of gambling (e.g. betting shops, casinos, amusement parks)	NGOs
Off-shore operators of gambling (e.g. online gambling such as sports betting, casino, bingo)	Individuals (with no tie to the industry)
Manufactures and distributors of gambling machines (e.g. fruit machines, gaming etc.)	Local/governmental authorities
Game designers (online and physical)	Regulators
Lottery operators	Academics
Social aspects and public relations organisations (SAPROs) (e.g. charities funded by the industry)	Licensing firms/consultants^a^
Trade associations	Any other entities falling outside the gambling industry definition

a Licensing firms/consultants have been omitted because in addition to representing industry, these firms will also represent local authorities and the police; therefore, it is very difficult to tease out to what extent they are representing industry interests in their responses.

A thematic analysis was undertaken by first reading and re-reading the industry responses to the inquiries to become familiarised with the data. Submissions were then descriptively coded. Codes were reviewed and organised into reoccurring ‘themes’ by comparing and exploring relationships between the codes. The development of themes was an iterative process and themes evolved as more responses were coded. Themes were organised into two overarching groups: (i) how the problem is framed and (ii) how the solution is framed. S.Bh. led the analysis. S.Bo. and K.S. familiarised themselves with the primary data and checked the coding for plausibility, validating the first author’s (S.Bh.) interpretation through an iterative process.

## Results

A total of 161 and 98 written responses were submitted to the HoL inquiry into the ‘Licensing Act 2003’ (2016/17) and the HoL inquiry into the ‘Social and Economic Impact of the Gambling Industry’ (2019/20), respectively. These figures exclude supplementary written responses. Of these, 19/161 responses (∼12%) were identified as alcohol industry responses (making up the second largest proportion of all responses) and 28/98 responses (∼29%) were identified as gambling industry responses (making up the largest proportion of all responses). A list of each industry respondent to the alcohol and gambling inquiry and a category breakdown are given in Supplementary files S2 and S3.

Responses varied in terms of which and how many questions they responded to; many responses referenced other responses also submitted to the same inquiry where their interests aligned and often had sections that were either very similar or identical.

Themes in arguments/strategies identified in the responses were organised into two overarching groups: how the problem is framed and how the solution is framed. A collection of quotes to illustrate each theme is provided in table 3. A more detailed version can be found in Supplementary file S4. Though themes in the arguments/strategies used by industry are presented here separately, they are linked and often used in tandem.

**Table 3:** Selected quotes to illustrate themes identified in industry responses to the inquiries

Framing of the problem
Most people drink/gamble responsibly
**‘It ignores the fact that again, millions of people go out in the UK and across London every week without incident and enjoy themselves, form new friendships, relax, get inspired and go home’ (Night Time industries Association, Alcohol Industry Trade Association)**
**‘As in other areas of regulation, it would be wrong to judge a whole sector on the actions of some outliers who maliciously or inadvertently are in breach of the rules’ (Responsible Affiliates in Gambling, SAPRO)**
The evidence of harms is overstated
**‘It is our perception that research is dominated by middle class academic thinking and it does not accommodate sufficiently a wide range of potentially difficult views from others’ (Bacta, Gambling Trade Association—Amusements Operators/Machine Manufacturers)**
Other actors are to blame
**‘There are no such rules and regulations stopping consumers from taking high interest credit facilities to buy luxury items or luxury consumer goods beyond the consumers’ affordability, likewise no obligations or systems enforced on retailers to ensure customers are aware of how much alcohol or tobacco is being purchased and consumed’ (Bet Victor Limited, Off-Shore Gambling Operator)**
**‘We also believe that the vast majority of alcohol related problems are created away from the on-trade where there is a trained Designated Premises Supervisor to supervise responsible drinking and the age of the individuals consuming alcohol … There are no such controls in the off-trade once alcohol has left the premises’ (Admiral Taverns, Pub Operator)**
**‘We believe that the primary reason for the prevalence of anti-gambling industry related sentiment in the UK is both the volume and the tone of gambling advertising in and around televised sports events’ (Rank Group, On-Shore Operator—Bingo & Casinos)**
The industry is part of the solution
**‘We are aware of criticism of industry participation in research but contend that the involvement of licensees involves a number of benefits (including better access to consumers and consumer data and greater engagement in harm reduction)’ (Hippodrome Casino Limited, Land based gambling operator)**

**Framing of the solution**

The industry is socially responsible
**‘The off-trade has led the way in the introduction of age verification schemes such as ‘Challenge 25’ and partnership schemes including Community Alcohol Partnerships. This has helped to significantly reduce the number of underage people purchasing alcohol’ (Association of Convenience Stores, Alcohol Trade association—On-Trade Retailers)**
Targeted and/or localised solutions are needed
**‘A higher Minimum Unit Price would push up the prices in shops for around half of all alcohol for consumers in England and Wales and impact on those on the lowest incomes. It is not a targeted measure, hitting all drinkers regardless of how responsible they consume alcohol and is unlikely to impact those the heaviest drinkers that are least responsive to price changes’ (Wine and Spirit Trade Association, Alcohol Trade Association)**
The problem is too complex to be solved by population measures
**‘Blanket approaches to control so called ‘super-strength’ products are ineffective in tackling complex alcohol-related issues and are in stark contrast to the collaborative and locally targeted initiatives that are broadly considered by the majority of stakeholders as the preferred approach’ (British Beer and Pub Association, Alcohol Trade Association)**
A population level response would be harmful
Harms to consumers
**‘Further restrictions on the on-trade could irreversibly damage the sector and further tip the balance to the off trade—where consumption is unregulated and therefore health risks to the consumer are greater’ (Campaign for Real Ale, SAPRO)**
**‘We feel that the UK regulators sometimes miss the very great contribution pubs, bars, restaurants and nightclubs, make to society, whether through jobs, investment or even simply the socialising that is enjoyed by millions on a weekly of not daily basis’ (Beds and Bars, on-trade alcohol retailer—pub operator)**
Harms to industry
**‘We can go about do-good projects to protect a tiny, but vocal element, of problem gamblers and the cottage industry that has grown up to support the same. However, in doing so we wreck our economy, put thousands out of work, decimate our High Streets and industries such as Racing who depend upon gambling to survive’ (Geoff Banks Sports Advisors, Online gambling operator (sports betting))**
Harms to the wider economy
**‘It is important to note that the Government’s Economic Impact Assessment highlighted that an MUP of 45p would cost the Treasury £200 m in lost revenue and also cost consumers an additional £1bn and, at a time of significant uncertainty for business and the Government, this could have a significant impact’ (Wine and Trade Spirit Association, Alcohol Trade Association—manufacturers)**

### The problem

#### Most people drink/gamble responsibly

Alcohol and gambling industry actors framed the problem of alcohol/gambling similarly, emphasising, incorrectly, that harms were only experienced by a minority of people who consume in ‘excess’, while the majority drink/gamble ‘responsibly’, though responsible consumption was undefined. Harms to the wider population or harms of ‘low level’ drinking/gambling were generally not stated, except occasionally in reference to crime by people who consume large quantities of alcohol, crime by people who need money to gamble, or money laundering in betting shops.

This reasoning was also extended to operators, with responses stressing that only a small minority operate irresponsibly. Some industry responses argued that they should not be penalised for a minority of individuals/operators who act irresponsibly.

Framing the problem as only affecting a minority of consumers or operators was often part of a bigger strategy to argue against population level regulation (see ‘framing of the solution’ section).

#### The evidence of harms is overstated

Most responses spoke about the potential harms of alcohol/gambling (the gambling inquiry specifically asked about the social and economic costs), quoting evidence or claiming that overall trends in alcohol consumption are falling, the prevalence of problem gambling is low, underage drinking/gambling is low and alcohol/gambling-related crime is low. Some gambling industry responses also claimed the UK performs well by international standards.

In addition, gambling industry submissions commonly pointed out potential flaws in gambling research—e.g. statistics being out of date or extrapolated from small samples—to cast doubt on the science.

A few submissions went as far as to imply researchers are biased, arguing ‘research is dominated by middle class academic thinking’ and more ‘neutral’ research is needed.

#### Other actors are to blame

Both the alcohol and gambling industry sought to shift blame to (i) other UCIs and (ii) other parts of their industries. Where harms caused by other UCIs were discussed, sometimes these were only tangentially related.

Where other parts of their industry were blamed, this involved on-trade alcohol retailers (i.e. pubs) deflecting the problem to the off-trade (i.e. supermarkets, off-licences) and on-shore gambling operators (i.e. casinos, betting shops) deflecting to off-shore (online) operators. In the alcohol inquiry, there was a specific question regarding off-trade alcohol consumption, where on-trade retailers took the opportunity to highlight that more alcohol is sold by the off-trade. They argued this facilitates binge drinking/pre-loading and that on-trade venues are safer to drink in, as they are supervised.

Similarly, on-shore gambling operators appeared to shift blame for gambling harms to off-shore gambling operators, where there are fewer safeguards and controls for vulnerable customers and gambling is ‘unsupervised’. In addition, some industry responses suggested that dislike for the gambling industry stems from excessive sports betting advertising.

This ‘shifting blame’ argument was generally used to oppose further regulation for the on-trade/on-shore actors, with restrictions on these actors considered to be disproportionate compared to the off-trade/off-shore ones. Some off-trade/off-shore actors appeared to anticipate this argument and pre-emptively defended themselves.

#### The solution

Industry actors favoured solutions to alcohol/gambling harms that were local and targeted and discouraged solutions that consisted of population level regulation. These proposed solutions appear to build directly on how industry actors framed the problem in their responses (i.e. only affecting a minority of irresponsible or ‘at risk’ consumers).

### Industry favoured solutions

#### The industry are part of the solution

In the majority of responses, both the alcohol and gambling industry presented themselves as part of the solution, emphasising the importance and advantages of working with industry stakeholders. Arguments used included: industry has access to data and consumers (for research), industry can provide expertise and partnership approaches between industry and local communities/government are most effective and fair. Some industry actors made reference to the negative connotations and possible conflict of interest associated with working with industry but argued that when done ‘appropriately’, operator input into solutions is beneficial.

#### The industry is socially responsible

As part of presenting themselves as part of the solution, industry actors also portrayed themselves as ‘socially responsible’. For example, some responses provided more than one page of examples of current industry-led initiatives.

Highlighting industry contributions to a safer gambling/drinking environment generally served to promote voluntary/self-regulation of industry, since industry argued that they had already proven their commitment to social responsibility.

#### A targeted and/or localised solution is required

Based on the framing of alcohol and gambling harms as a problem for only a minority of individuals, industry responses suggested that targeted/localised solutions will help those who need it most, without compromising the enjoyment of responsible consumers or penalising responsible operators.

Gambling industry responses mainly promoted investment in treatment services, interventions that identify and target ‘problem’ gamblers (e.g. technology to aid self-exclusion from gambling websites or affordability checks), or youth education campaigns about ‘safe’ and ‘responsible’ gambling, etc. Alcohol industry responses similarly supported local solutions via schemes such as Community Alcohol Partnerships.

A small number of specific population level interventions were supported, e.g. a voluntary ‘whistle-to-whistle’ pre-watershed TV advertising ban (i.e. gambling advertising during live sports broadcast before 9 pm), increasing the minimum age for playing gambling products/lotteries to 18 and ‘responsible’ drinking/gambling campaigns.

### Industry opposed solutions

Most alcohol and gambling industry responses were opposed to population level measures (apart from the above). For example, many of the alcohol industry responses were opposed to a minimum price at which one unit of alcohol can be sold (minimum unit pricing), late night levies (fees for premises serving alcohol after a certain time), bans on super strength alcohol and stricter licensing regimes. Many gambling industry responses were opposed to the introduction of a statutory levy, further limits on advertising, restrictions on the number of gambling machines allowed in a venue and a ban on the use of debit cards on gambling machines.

However, not all responses were in harmony. There were exceptions, for instance, typically where implementation of the population level measure would unlikely have an impact on the respondent’s particular aspect of the industry.

A few non-sports betting gambling operators were in favour of a ban or harsher restrictions on sports betting advertising (see ‘other actors are to blame’ section).

#### The problem is too complex to be solved by population level measures

A common strategy used in alcohol and gambling industry responses to oppose population level measures was to claim that it is difficult to assign causation of ‘problem drinking/gambling’ to the industry, since there are many complex reasons why someone may drink/gamble in excess. This argument was more common in gambling industry responses.

When presenting the problem as ‘complex’, industry actors often framed population level solutions as ‘too simplistic’ to be able to tackle issues as ‘complex’ as problem drinking/gambling. Instead, targeted/local solutions (i.e. the industry-preferred solutions above) were framed as preferential. This argument was more common in alcohol industry responses.

#### A population level response would be harmful

Alcohol and gambling industry actors also commonly listed the potential harms of implementing population level measures. A variety of different potential harms were given, sometimes backed up with evidence and sometimes without providing any evidence. Harms suggested by the industry can be split into three categories: (i) harms to the consumer, (ii) harms to a responsible industry and (iii) harms to the wider economy.

Both industries claimed population measures would reduce consumer choice and would push consumers to the off-trade/off-shore, where drinking/gambling is unsupervised. Both industries also highlighted how regulation ignores the benefits of their industries, e.g. the cultural contribution of pubs/bars, social aspects and enjoyment. The gambling industry also suggested that population measures may push gamblers into riskier behaviours and suggested that forcing the gambling industry to participate in a mandatory levy would reduce their incentive to fund research, education and treatment. The alcohol industry suggested minimum unit pricing would have a disproportionate impact on people with a low income.

Among harms suggested to a responsible industry, negative impacts on businesses/jobs and in the case of the alcohol industry, impacts on the on-trade, were frequently raised. These were presented as arguments against population level measures. A small number of alcohol and gambling industry responses made similar arguments against the impact of population level regulation, this time considering the wider economy. This included commenting on the impact of restrictions on other industries dependent on the alcohol or gambling industry and losses to the government in tax revenue.

## Discussion

Arguments used by both industries were mutually-reinforcing; framing the problem as one that only affects a small minority of people, emphasising individual responsibility and shifting blame to other industry actors, all of which implicitly frames population level regulation as redundant and favours industry-preferred targeted/local solutions. Striking similarities in the framing and arguments used by both alcohol and gambling industry actors supports findings from other studies that UCIs use the same corporate political strategies to shape the narrative in favour of their commercial interests.^1^

Industry view of harms from alcohol and gambling was narrow. Framing the problem as only affecting a small minority of people (while the majority of people enjoy recreational drinking or gambling ‘responsibly’) and emphasising of ‘individual responsibility’ is consistent with previous research about the alcohol^3^^,^^4^^,^^11^^,^^12^^,^^17^ and tobacco industries,^10^ and inconsistent with what is known about harms from alcohol and gambling. Harms occur on a continuum, i.e. there is no safe, or completely risk free, level of consumption.^18^^,^^19^ This framing seeks to distance industry actors from problems caused by consumption and shift blame for alcohol and gambling harm to those who (mis)use these products^4^ while ignoring the contribution of UCIs in shaping the environments in which harms occur (e.g. the addictive nature of UCI’s ‘unhealthy’ products and norms perpetuated by pervasive alcohol and gambling presence in media and persistent marketing).^20^^,^^21^

Both industries also sought to shift blame to other actors in the same industry. Most commonly this was from on-trade to off-trade alcohol retailers or on-shore to off-shore gambling operators. In addition, there was a general dislike of excessive sports betting advertising by other gambling industry actors. This is a noteworthy finding as public health literature tends to refer to industry views and framing as unanimous; however, arguments used by subsectors sometimes diverge, dependent on whether proposed regulation will affect them differentially. This warrants further research, because a fragmented industry could provide a potential avenue for public health policy makers to make a case for regulation in the interest of public health.^22^

Another novel finding was the doubt cast on gambling research and researchers by the gambling industry responses. The gambling industry exploited the ‘dearth’ of gambling statistics, lack of current research (the last British Gambling prevalence survey was undertaken in 2010,^23^ for instance) and industry perceived bias of researchers in their responses. This is reminiscent of general UCI tactics which seek to point out flaws or cast doubt on research as a strategy to downplay their harms, or as an excuse not to act until more research can be carried out.^24^

By concentrating on harms to small minority of individuals, industry legitimise their preferred solutions to these harms,^25^ i.e. self-regulatory initiatives, education programmes and local/targeted solutions, which rely on the agency of individuals and promote ‘personal responsibility’. Framing of evidence-based population level solutions as ‘too simple’ to solve ‘complex’ problems, despite obvious flaws in this reasoning (as targeted solutions can also be considered simple), and making (often unsubstantiated) claims regarding the unintended consequences of population level seek to distance industry as major contributors to gambling/alcohol harms and undermine scientific consensus over population level measures.^13^ Taxation, restricting marketing and restricting availability have a good evidence base^26^ while industry-led initiatives are have been criticised for serving industry agendas (normalising harmful products and shifting responsibility to individuals who consume these products).^25^^,^^27^ Analysis of responsible drinking adverts and campaigns shows that industry-vested interests lead to adverts which are purposefully vague, do little except create the illusion that industry is socially responsible^28^ and may even be harmful by acting as pictorial cues to drink.^29^

Many public health researchers have questioned the legitimacy of UCI involvement in policy making.^1^ Policy makers and researchers should be careful not to take a narrow view of gambling and alcohol harm, which plays into industry narratives, and recognise the contribution of commercial actors and policy makers to the environment in which harms happen.^30^

A strength of this study is that it is a comparative analysis of two UCIs. By comparing the framings and arguments used by the alcohol and gambling industry, it provides evidence that UCIs largely use the same strategies to promote their interests and identifies a few subtle differences in their approach. The sample size (*n* = 47 total industry responses) was also relatively large, providing weight to findings. The two HoL inquiries were also conducted in a close time period (carried out in 2016 and 2019), had a similar subject matter and followed a similar format, making them suitable for comparison. In addition, there are relatively few studies analysing the actions of the gambling industry, thus this study adds to the evidence base of an emerging and topical field of research.

This study has some limitations. As Rinaldi et al.^3^ pointed out, it is not possible to know the motivation for the framings and arguments used by industry actors from this type of research, and neither can the outcome of this framing on subsequent policy decisions. Future studies comparing industry responses with public health, NGO and/or academic stakeholder responses to the inquiries would therefore be useful in giving authority to conclusions that industries undertake such framing to serve commercial interests.

It was beyond the scope of our project to assess how UCI lobbying efforts compare with lobbying efforts in areas that do not pertain to public health, but it would be an interesting area for further research.

Though the two inquiries analysed were similar in format and subject, they were not exactly the same in the topic areas and questions asked. This presented a risk that differences in themes/arguments identified across the two industries’ responses may simply reflect differences in the questions asked. However, given the similarities identified in the alcohol and gambling industry submissions, this seems unlikely to have been the case.

## Conclusions

Comprehensive thematic analysis of alcohol and gambling industry responses to two HoL inquiries has shown that these industries largely use the same framing, arguments and strategies to shape the discourse around harms and solutions in their commercial interests. This study had novel findings around alcohol and gambling industries shifting blame for harms to other actors in their same industries, and exploitation of a supposed ‘dearth’ of evidence as a strategy to downplay harms or delay action in the field of gambling.

This study supports calls for a reframing of gambling harms which takes into account harms experienced by society and recognises contributions of commercial actors and policy makers to the environment in which harms happen.^30^

Understanding and communicating the commonalities in strategies used by industries may help to inform more effective and unified public health responses across all UCIs.

## Supplementary Material

ckad018_Supplementary_Data

## Data Availability

The data that support the findings of this study are openly available; responses to the inquiries into the Alcohol ‘Licensing Act 2003: post-legislative scrutiny’ and ‘Social and Economic Impact of the Gambling Industry’ are available at https://old.parliament.uk/licensing-act-committee/ and https://committees.parliament.uk/committee/406/gambling-industry-committee/, respectively. Key points The alcohol and gambling industries largely use the same framing, arguments and strategies to shape the discourse around harms and solutions in their commercial interests. Framing the problem as one of ‘individual responsibility’, only experienced by a minority, promotes industry agendas; policy makers and researchers should therefore be careful not to take a narrow view of gambling and alcohol harm. Policy makers should be made aware of industry strategies to avoid undue industry influence on policy decisions and presented with evidence on past successes and failures in countering corporate political activity and conflicts of interest in policymaking across unhealthy commodity industries.
