# Supplementary material for: A comparative study of industry responses to government consultations about alcohol and gambling in the UK
Source: Eur J Public Health. 2023 Feb 28;33(2):305–11. doi: 10.1093/eurpub/ckad018 (PMC10066481; doi:10.1093/eurpub/ckad018)
Supplement: ckad018_Supplementary_Data [file ckad018_supplementary_data.zip › ckad018_Supplementary_Data/ejph-2022-06-om-0324-File002.docx]

Supplementary file 1 Definitions of the Alcohol and Gambling industries

**Alcohol industry:**

In this study, we will use the World Health Organisation (WHO) definition of the alcohol industry, which has been adopted by Public Health England (PHE)[1] and explained in detail by the Institute of Alcohol studies (IAS)[2].

*The alcohol industry includes* ***“manufacturers of alcoholic beverages, wholesale distributors, major retailers and importers that deal solely and exclusively in alcohol beverages, or whose primary income comes from trade in alcohol beverages.***

***In addition, ‘alcohol industry’ includes business associations or other non-State actors representing, or funded largely by, any of the aforementioned entities***^ꝉ^***, as well as alcohol industry lobbyists and commercial interests in alcohol beverage trade other than above when the interaction … can be linked to their interests in alcohol beverage trade”.***

**Gambling industry:**

Despite an increase in gambling industry research, it was hard to find a formally agreed definition. This may, in part, be due the structure of the gambling industry changing rapidly in recent times.

Therefore, the above definition has been adapted and applied to the gambling industry:

*The gambling industry includes* ***operators of gambling (both on-shore e.g. physical shops/casinos/amusement parks and off-shore e.g. online versions of the latter, and parent companies), operators of lotteries and physical and digital gambling infrastructure (i.e. designers/providers of online games and manufactures and distributors of gambling machines).***

***In addition, ‘gambling industry’ includes business associations or other non-State actors representing, or funded largely by, any of the aforementioned entities***^[[1]](#footnote-1)^***, as well as gambling industry lobbyists and commercial interests in gambling other than above when the interaction can be linked to their interests in the gambling industry.***

1. Public Health England. Principles for engaging with industry stakeholders. 2019. https://www.gov.uk/government/publications/principles-for-engaging-with-industry-stakeholders/principles-for-engaging-with-industry-stakeholders. Accessed 20 Aug 2021.

2. Institute of Alcohol Studies. The alcohol industry: An Overview. 2016. http://www.ias.org.uk/.

3. The Institute of Alcohol Studies. The Alcohol Industry: Social and political activities. 2020. www.ias.org.uk.

1. ‘*Business associations or other non-State actors representing, or funded largely by, any of the aforementioned entities’ will include collective bodies such as trade associations and social aspects and public relations organisations (SAPROs) e.g. charities funded by the industry e.g. Drinkaware or BeGambleaware. These bodies tend to act on industry’s behalf in matters such as tax, marketing and regulation, research etc.*[3] [↑](#footnote-ref-1)
