# Supplementary material for: A comparative study of industry responses to government consultations about alcohol and gambling in the UK
Source: Eur J Public Health. 2023 Feb 28;33(2):305–11. doi: 10.1093/eurpub/ckad018 (PMC10066481; doi:10.1093/eurpub/ckad018)
Supplement: ckad018_Supplementary_Data [file ckad018_supplementary_data.zip › ckad018_Supplementary_Data/ejph-2022-06-om-0324-File003.docx]

Supplementary file 2 List of all Alcohol industry responses to the HoL inquiry into the ‘Licensing Act 2003’ (2016/17)

| Responder | Type of industry stakeholder |
| --- | --- |
| Admiral Taverns | On-Trade Retailer (Pub operator) |
| Association of Convenience Stores | Trade association (Off-Trade Retailer) |
| Association of Licensed Multiple Retailers | Trade association (Off-Trade Retailer) |
| Beds & Bars | On-Trade Retailer (Pub operator) |
| British Beer & Pub Association | Trade association (Manufacturers of Alcohol & Pub operator) |
| British Hospitality Association | Trade association (On-Trade Retailer) |
| British Retail Consortium | Trade association (On-Trade Retailer) |
| Campaign for Real Ale | SAPRO |
| Deltic Group | On-Trade Retailer (Nightclub operator) |
| Fabric Life Limited | On-Trade Retailer (Nightclub & Pub operator) |
| Federation of Wholesale Distributors | Trade association (Wholesale Alcohol distributors) |
| National Federation of Retail Newsagents | Trade association (Off-Trade Retailer) |
| Night Time Industries Association | Trade association (On-Trade Retailer) |
| Punch Taverns | On-Trade Retailer (Pub operator) |
| Sainsbury’s | Off-Trade Retailer (Supermarket) |
| Scotch Whisky Association | Trade association (Manufacturers of Alcohol) |
| Society of Independent Brewers | Trade association (Manufacturers of Alcohol) |
| Wine and Spirit Trade Association | Trade association (Manufacturers of Alcohol) |
| Working Men’s Club and Institute Union Limited | On-Trade Retailer (Nightclub operator) |
