# Supplementary material for: A comparative study of industry responses to government consultations about alcohol and gambling in the UK
Source: Eur J Public Health. 2023 Feb 28;33(2):305–11. doi: 10.1093/eurpub/ckad018 (PMC10066481; doi:10.1093/eurpub/ckad018)
Supplement: ckad018_Supplementary_Data [file ckad018_supplementary_data.zip › ckad018_Supplementary_Data/ejph-2022-06-om-0324-File004.docx]

Supplementary file 3 List of all Gambling industry responses to the HoL inquiry into the ‘Social and Economic Impact of the Gambling Industry’ (2019/20)

| Responder | Type of industry stakeholder |
| --- | --- |
| Association of Convenience Stores | Trade association (Lottery Retailer) |
| Bacta | Trade association (Amusements and high street gaming Operators and Machine Manufacturers) |
| Betting and Gaming Council | Trade association (All types of Gambling) |
| BetVictor Limited | Off-Shore Operator (Sports Betting) |
| British Beer and Pub Association | On-Shore Operator/Trade association (Alcohol) |
| British Horseracing Authority | Trade association (Sports Betting) |
| Camelot UK Lotteries Ltd | Lottery operator |
| English Football League^[[1]](#footnote-1)^ | Other |
| European Lotto Betting Association | Trade association (Other) |
| Federation of Racecourse Bookmakers | Trade Association (Sports Betting) |
| Flutter Entertainment Plc | On & Off-Shore Operator (All Types of Gambling) |
| BeGambleAware | SAPRO |
| GamCare | SAPRO |
| Gauselmann Group | On-Shore Operator (Casinos and other gaming venues) & Machine Manufacturer & Game designers (Online & Physical) |
| Geoff Banks Sports Advisors | Off-Shore Operator (Sports Betting) |
| GVC Holdings Plc | On & Off-Shore Operator (All Types of Gambling) |
| Hippodrome casino | On-Shore Operator (Casino) |
| National Casino Forum | Trade association (Casinos/Bingo/Amusement Parks) |
| National Lottery Distributors | Other |
| Novomatic UK Ltd | On-Shore Operator (Casinos, Sports Betting) & Gaming Machine Manufacturer |
| People's Postcode Lottery | Lottery operator |
| Rank Group | On-Shore Operator (Bingo & Casino) |
| Responsible Affiliates in Gambling (RAiG) | SAPRO |
| Sky | Other |
| Sky Betting and gaming | Off-Shore Operator (Sports Betting) |
| The Bingo Association | Trade association (Bingo) |
| The Lotteries Council | Lottery operator |
| William Hill Plc | On & Off-Shore Operator (Sports Betting) |

1. Included as some of their cups are sponsored/funded by Sky Bet (gambling operator) [↑](#footnote-ref-1)
