# Supplementary material for: A comparative study of industry responses to government consultations about alcohol and gambling in the UK
Source: Eur J Public Health. 2023 Feb 28;33(2):305–11. doi: 10.1093/eurpub/ckad018 (PMC10066481; doi:10.1093/eurpub/ckad018)
Supplement: ckad018_Supplementary_Data [file ckad018_supplementary_data.zip › ckad018_Supplementary_Data/ejph-2022-06-om-0324-File005.docx]

Supplementary file 4 Selected quotes to illustrate themes identified in responses

| Overarching Frames | Arguments/strategies | Selected quotes |
| --- | --- | --- |
| Framing of the Problem | **Most people drink/gamble responsibly** |  |
|  | - And only a minority are problem drinkers/gamblers | *“It ignores the fact that again, millions of people go out in the UK and across London every week without incident and enjoy themselves, form new friendships, relax, get inspired and go home” (Night Time industries Association, Alcohol Industry Trade Association)*  *“I respectfully point out that for many millions of people gambling is a highly enjoyable pastime and gives meaning and purpose to many sporting events for the same. Yes, there are those who ‘tip over the edge’ – who go too far. But this is a tiny minority of those who gamble” (Geoff Banks Sports Advisors, online gambling operator (sports betting))* |
|  | - Most pubs/betting shops/casinos etc. operate responsibly (and only a minority operate irresponsibly) | *“Our view is that there are sufficient provisions for dealing with “problem premises” without the need to burden licence holders with a blanket approach” (Deltic group, Nightclub operator)*  *“As in other areas of regulation, it would be wrong to judge a whole sector on the actions of some outliers who maliciously or inadvertently are in breach of the rules” (Responsible Affiliates in Gambling, SAPRO)* |
|  | - Industry cannot be held responsible for the minority who do not drink/gamble responsibly | *“More can be done to ensure that a distinction between personal responsibility and a businesses responsibility is made in both the Guidance and local licensing policies. At the moment, premises operators face enforcement action, when matters should correctly be ascribed to the actions of an individual or individuals. We can point to examples where action appears to be taken against us as premises licence holders, where in fact our publicans are just as much victims of criminal action as anyone else” (Punch Taverns, Pub operator)*  *“Gambling consumers also need to accept a level of responsibility for their own actions and behaviours” (Hippodrome Casino Limited, Land based gambling operator)* |
|  | **The evidence of harms is overstated** |  |
|  | - Alcohol consumption/problem gambling has fallen/is low | *“Alcohol consumption and alcohol related harm is in long term decline. The proportion of people who drank in the last week fell from 64% in 2005 to 58% in 2013 and the proportion of young people that had binged in the last week has fallen from 29% in 2005 to 18% in 2013” (Federation of Wholesale Distributors, Trade Association)*  *“Reported rates of gambling by children have declined significantly in recent years. Between 2011 and 2017, past-week gambling by 11-to15-year-olds fell from 23% to 12%. Gambling by children on age restricted products appears to be relatively low – much lower for example than for consuming alcohol…” (Betting and Gaming Council, Gambling Trade Association)*  *“Great Britain has (so far as we can tell) low levels of illegal gambling; relatively low levels of problem gambling by international standards” (Betting and Gaming Council, Gambling Trade Association)* |
|  | - Gambling research is flawed | *“While we are sure that the seemingly small pool of researchers active in this field are well qualified, it is important for any research bank that the research is conducted by a neutral and diverse body of researchers, holding a range of backgrounds, qualifications, specialisms and interests. To date that does not appear to be the case”* *(Novomatic UK Ltd, Land based gambling operator)*  *“The absence of a systematic framework to measure costs or harms has resulted in a dearth of reliable data and the generation of some questionable research studies” (Hippodrome Casino Limited, Land based gambling operator)* |
|  | **Other actors are to blame** |  |
|  | - Deflect to other UCIs | *“There are no such rules and regulations stopping consumers from taking high interest credit facilities to buy luxury items or luxury consumer goods beyond the consumers’ affordability, likewise no obligations or systems enforced on retailers to ensure customers are aware of how much alcohol or tobacco is being purchased and consumed” (Bet Victor Limited, Off-Shore Gambling Operator)* |
|  | - On-trade alcohol retailers deflect the problem to off-trade alcohol retailers | *“We also believe that the vast majority of alcohol related problems are created away from the on-trade where there is a trained Designated Premises Supervisor to supervise responsible drinking and the age of the individuals consuming alcohol again supported by the excellent Challenge 21 schemes prevalent in the on-trade. There are no such controls in the off-trade once alcohol has left the premises” (Admiral Taverns, Pub Operator)*  *“The incentive to ‘pre-load’ increases as the price difference widens between alcohol bought from retail distributors to those in licensed establishments, which in turn encourages drinking prior to going out. The British Hospitality Association would like authorities to make full use of powers they already have at their disposal to control the off-trade, before consideration of the granting of new powers or adding further regulation”(British Hospitality Association, Alcohol Trade association - On-Trade Retailers)* |
|  | - On-shore gambling operators deflect the problem to off-shore gambling operators/sports betting | *“There are well established safeguards and controls in the nonremote sector that the Commission have put in place to protect the vulnerable from harm... These measures however have not been put in place for the remote sector which meant that online and mobile operators can develop games without controls that would help to protect the vulnerable and ensure that those games are fair and safe” (Gauselmann Group, Land-based gambling operator and machines manufacturer/supplier)*  Gambling industry deflect to sports betting:  *“we believe that the primary reason for the prevalence of anti-gambling industry related sentiment in the UK is both the volume and the tone of gambling advertising in and around televised sports events” (Rank Group, On-Shore Operator - Bingo & Casinos)*  *“A significant number of operators would not object to a reduction or elimination of TV advertising” (National Casino Forum, Gambling Industry Trade association - Casinos/Bingo).*  On-shore/on-course sports betting deflect to off-shore betting:  *“Finally, our business model is not based on incentivisation for increased betting activity. Unlike the off-course and on-line industry, we do not offer bonuses or gimmicks to staff who encourage punters to continuously bet, nor do we advertise” (Federation of Racecourse Bookmakers, Gambling Trade Association – Sports betting)* |
|  | - Off-trade alcohol retailers/off-shore gambling operators deflect to the on-trade/on-shore | *In many ways online gaming/gambling regulations and their enforcement are more sophisticated than those applicable to retail operators due to the multiple additional levels of control” (European Lotto Betting Association, Gambling Trade Association).* |
| Framing of the solution | **The industry is part of the solution** |  |
|  | - In terms of research | *“Further, we acknowledge and agree that independence is important. However, if done appropriately, we believe that operators can have an important role to play given our expertise, access to data, and our understanding of that data and its context” (Sky Betting and Gaming, Off-shore Gambling operator)*  *“We are aware of criticism of industry participation in research but contend that the involvement of licensees involves a number of benefits (including better access to consumers and consumer data and greater engagement in harm reduction)” (Hippodrome Casino Limited, Land based gambling operator)* |
|  | **The industry is socially responsible** |  |
|  | - And therefore there is no need for further regulation | *“The off-trade has led the way in the introduction of age verification schemes such as ‘Challenge 25’ and partnership schemes including Community Alcohol Partnerships. This has helped to significantly reduce the number of underage people purchasing alcohol” (Association of Convenience Stores, Alcohol Trade association - On-Trade Retailers)*  *“Important restrictions on advertising have been implemented in recent years through self-regulation and the actions of regulatory bodies without the need for legislation. We believe that this approach offers the greatest opportunity for agile responses to concerns while allowing benefits to be retained for recreational gamblers” (Betting and Gaming council, Gambling Industry Trade Association).*  In reference to a mandatory levy on the gambling industry:  *“The effectiveness of the voluntary system has increased over recent years. Voluntary commitments by the largest operators to increase funding suggests that the voluntary scheme can be relied upon to achieve not simply current funding requirements but also a substantial and sustained increase in funding requirements” (Betting and Gaming Council, Gambling Trade Association)* |
|  | - In terms of partnerships | *“The WSTA believes that the most effective approach to tackling problems that are faced in local communities is to develop a partnership that treats retailers as part of the solution rather than just as part of the problem. This has occurred through Community Alcohol Partnerships in Great Yarmouth and Hastings which have look to tackled street drinking as part of its activities. This has been achieved without the need for further regulation” (Wine and Spirit Trade Association, Alcohol Trade Association)* |
|  | **Targeted and/or localised solutions are needed** |  |
|  | - So we don’t penalise those who drink/gamble responsibly | *“A higher Minimum Unit Price would push up the prices in shops for around half of all alcohol for consumers in England and Wales and impact on those on the lowest incomes. It is not a targeted measure, hitting all drinkers regardless of how responsible they consume alcohol and is unlikely to impact those the heaviest drinkers that are least responsive to price changes” (Wine and Spirit Trade Association, Alcohol Trade Association)* |
|  | **The problem is too complex to be solved by population measures** |  |
|  | - Difficult to assign causation to the industry | *“The socio-economic costs of gambling are difficult to determine in isolation due to the close association of problem gambling with a range of other issues including poor mental health and substance abuse amongst others. Attempting to attribute the costs associated with such problematic behaviours to a single industry/set of products is unhelpful and even counterproductive as it often conceals issues of larger scale” (European Lotto Betting Association, Gambling Trade Association)* |
|  | - And cannot be solved by simple blanket approaches | *“Blanket approaches to control so called ‘super-strength’ products are ineffective in tackling complex alcohol-related issues and are in stark contrast to the collaborative and locally targeted initiatives that are broadly considered by the majority of stakeholders as the preferred approach” (British Beer and Pub Association, Alcohol Trade Association)*  *“Taxation serves to generate revenue for government. It is often portrayed as a ‘silver bullet’ in tackling alcohol-related harm. Tackling alcohol-related harm is a complex issue and complex issues are not typically addressed by simple solutions. We know from experience of other countries, for example in Scandinavia, that having high levels of taxation do not necessarily lead to lower levels of heavy episodic drinking” (Scotch Whiskey Association, Alcohol Trade Association)* |
|  | **A population level response would be harmful** |  |
|  | *Harms to consumers:* |  |
|  | - Erode consumer choice | In reference to a ban on high strength beers:  *“As the strength of beers has increased over time, bans that limit beers with as low ABV as 5-6% can severely impact on consumer choice” (Campaign for Real Ale, SAPRO)* |
|  | - Regulation ignores benefits | *“We feel that the UK regulators sometimes miss the very great contribution pubs, bars, restaurants and nightclubs, make to society, whether through jobs, investment or even simply the socialising that is enjoyed by millions on a weekly of not daily basis” (Beds and Bars, on-trade alcohol retailer – pub operator)* |
|  | - Consumers will engage in riskier behaviours | In reference to current restrictions to the number of machines allowed in casinos:  *“It is also possible that the current, aforementioned restrictions have a number of negative unintended consequences: 1. Encouraging persistent play (through a fear of losing one’s place at a machine); 2. Increasing staking levels (through absence of choice of lower stakes games)” (Rank Group, Land based gambling operator (Bingo and Casino))*  In reference to whether gambling operators should have a legal duty of care to customers:  *“Introducing a legal standard may also create a ‘risk free’ betting environment where more customers who wouldn’t otherwise trying gambling, do so on the illusion that no matter what happens, the operator has a legal duty to protect the customer come what may” (BetVictor Limited, off shore gambling operator)* |
|  | - Impact on people with a low income | In reference to minimum unit pricing of alcohol:  *“The beer and pub sector is committed to reducing the harmful use of alcohol. However, pricing and taxation are blunt instruments to achieve this, penalising those on low incomes and responsible drinkers” (British Beer & Pub Association, Alcohol Trade Association)* |
|  | - Pubs/gambling venues are supervised | *“Further restrictions on the on-trade could irreversibly damage the sector and further tip the balance to the off trade – where consumption is unregulated and therefore health risks to the consumer are greater” (Campaign for Real Ale, SAPRO)* |
|  | - Industry have less incentive to invest in research/ treatment etc. | In reference to a mandatory levy on the gambling industry:  *“the shift from a voluntary scheme to a mandatory one may result in a diminution of engagement and interest between the industry and organisations carrying out work in research, education and treatment (which we observe anecdotally appears to have happened in certain jurisdictions where statutory schemes are in place). In short, there may be advantages to a system built on intrinsic motivation rather than coercion” (Hippodrome Casino Limited, Land based gambling operator)* |
|  | *Harms to industry* |  |
|  | - Impact on businesses (e.g. closures, job losses etc.) | *“We can go about do-good projects to protect a tiny, but vocal element, of problem gamblers and the cottage industry that has grown up to support the same. However, in doing so we wreck our economy, put thousands out of work, decimate our High Streets and industries such as Racing who depend upon gambling to survive” (Geoff Banks Sports Advisors, Online gambling operator (sports betting))* |
|  | *Harms to the wider economy* |  |
|  | - Impact on tax revenue | *“Of course the Government itself has in effect shot itself in the foot. The country needs business to fund taxation. The removal of the betting shops has left a giant fiscal gap” (Geoff Banks Sports Advisors, Off-Shore Gambling operator -Sports Betting)*  *“It is important to note that the Government’s Economic Impact Assessment highlighted that an MUP of 45p would cost the Treasury £200m in lost revenue and also cost consumers an additional £1bn and, at a time of significant uncertainty for business and the Government, this could have a significant impact” (Wine and Trade Spirit Association, Alcohol Trade Association – manufacturers)* |
|  | - Impact on Highstreet | *“Limiting the number of off-licences in an area also acts as a disincentive for new store openings and will mean more empty units on the high street and more under invested stores. Currently vacancy rates on UK high streets remain high at 12.4%” (Association of Convenience Stores, Alcohol Trade association - Off-Trade Retailer)* |
